# Influence of boundary layer conditions and surface properties on bacterial interaction with indoor built surfaces

**DOI:** 10.3389/fped.2026.1895305

**Published:** 2026-08-19

**Authors:** Dahae Seong, Shamia Hoque

**Affiliations:** 1 Graduate School of Particulate Matter Management, Kangwon National University, Gangwon-do, Republic of Korea; 2 Department of Civil and Environmental Engineering, College of Engineering and Computing, University of South Carolina, Columbia, SC, United States

**Keywords:** bacterial adhesion, indoor air quality, infection transmission, microbial detachment, near-surface airflow, particle resuspension, surface contamination, surface wettability

## Abstract

Indoor surface contamination is a significant concern in high-activity spaces such as childcare facilities, where microbial particles can be easily transmitted through direct surface touch or resuspension into indoor air. This study investigated *Corynebacterium* sp. attachment and detachment behaviors on four representative indoor surface materials (carpet, wood, metal and glass) and characterized near-surface airflow fields using batch attachment experiment, centrifugal detachment experiment, and particle image velocimetry (PIV). Surface characteristics: roughness, contact angle, and porosity were measured. The attachment fraction increased with exposure time for all tested surfaces, with metal (∼0.59), followed by wood (∼0.48) and glass (∼0.36). Carpet generated two microenvironments: an outer-fiber with higher-velocity region and an inter-fiber with lower-velocity shelter region. Glass showed the most uniform near-surface flows while wood generated the most spatially heterogeneous velocity fields due to its topography and pore structure. Overall, these results indicate that microbial attachment and detachment on indoor surface materials are governed by complex interplay of surface physicochemical properties, contact time, and geometry-dependent conditions, with no single factor acting alone. Smooth, non-porous surfaces could reduce initial adhesion and provide more predictable resuspension behavior in high-touch and high-activity indoor spaces.

## 1 Introduction

Indoor surface contamination has become an important public health concern because contaminated fomites can contribute to the transmission of infectious microorganisms through direct contact, resuspension, and subsequent inhalation or ingestion (1, 2). Humans live within a microbial cloud that reflects the individuals who predominantly occupy a space as a microbial bio-fingerprint (3). However, when pathogenic microorganisms are present, contaminated indoor surfaces serve as reservoirs that facilitate disease transmission, particularly in densely occupied environments such as childcare facilities and schools (4). Young children are especially vulnerable because of their developing immune systems, frequent hand-to-face contact, and prolonged interaction with shared indoor surfaces highlighting the importance of controlling contamination on high touch surfaces (5–7).

Previous studies have shown that frequently touched surfaces in childcare facilities, elementary schools, and hospitals are potential reservoirs for respiratory and enteric viruses such as influenza A/B virus, rhinovirus, and norovirus (8–13) and bacterial species such as *E. coli*, *Corynebacterium* sp. and *Pseudomonas* sp. (14–16). Commonly contaminated surfaces in these studies include door handles, faucet handles, desktops, and other wooden furnishings. Door handles are often identified as highly contaminated surfaces, while water-contact surfaces such as faucet handles and drinking fountain toggles frequently exhibit elevated bacterial contamination (9). These contaminated surfaces may subsequently contribute to secondary transmission through touch or through resuspension.

Microbial attachment to indoor surfaces is influenced by multiple physicochemical properties, including roughness, wettability, porosity, surface free energy, and surface chemistry. Surface flow conditions can enhance microbial retention or create conditions for easier removal. Seong et al. (17) showed that particle movement in the near-surface region is governed by both surface material properties and aerodynamic conditions. Studies have reported that microbial adhesion may increase on moderately hydrophobic surfaces with water contact angles near 90° (18) while bacterial detachment is generally more difficult from hydrophobic surfaces than from hydrophilic surfaces (19). Microbes on non-porous surfaces have been shown to transfer easily from fomites to fingers (20) with lower microbial contamination reported for non-wood and non-porous surfaces (21). However, no single surface characteristic independently determines microbial attachment or detachment behavior. Microbial adhesion and detachment processes are complex interplays among wettability, roughness, porosity, surface free energy, and particle properties. The relative importance of hydrophobicity may diminish when additional factors such as surface charge, roughness, and interfacial interactions are considered (22–24). Consequently, microbial adhesion and removal on indoor surfaces are governed by complex interactions among surface physicochemical properties, microbial characteristics, and external forces.

Previous studies have shown that resuspension behavior varies substantially depending on surface material, with textile surfaces exhibiting greater resuspension potential than hard surfaces (25–27). Near-surface airflow can enhance particle resuspension by amplifying the effects of human walking (26). Even under mechanically ventilated conditions, microbial concentrations near the floor are often governed more strongly by occupant activity than by ventilation airflow itself (28).

Although microbial adhesion and resuspension in indoor environments have been widely investigated, the coupled roles of surface characteristics and near-surface conditions remain insufficiently understood. In particular, limited information is available regarding how airflow structures near real indoor materials influence microbial transport, retention, and detachment. Therefore, the present study investigated *Corynebacterium* sp. attachment and detachment behaviors on representative indoor surface materials including carpet, wood, metal, and glass. *Corynebacterium* sp. is one of the predominant indoor bacteria, and certain species within this genus have been reported to cause respiratory and skin infections in children (16). Therefore, in this study, *Corynebacterium* sp. was selected as a model bacterium to investigate bacterial adhesion and detachment behavior due to its indoor prevalence and potential infection risks. Batch attachment experiments, centrifugal detachment tests, and particle image velocimetry (PIV) measurements were conducted to characterize microbial adhesion behavior and near-surface transport processes. In addition, a one-dimensional convection-diffusion model was applied to evaluate the influence of near-wall airflow on bacterial transport. The findings of this study provide mechanistic insight into microbial attachment and resuspension on indoor surfaces and may contribute to the development of improved surface hygiene and indoor environmental management strategies.

## 2 Materials and methods

### 2.1 Experimental design

Two types of experiments were conducted in this study. First, batch attachment and centrifugal detachment tests were performed to evaluate bacterial attachment and detachment temporally. Then, PIV (Particle Image Velocimetry) experiments were performed to characterize near-surface airflow and bacterial particle transport over real-world surfaces.

### 2.2 Bacterial preparations

*Corynebacterium* sp. strain used in this study (ATCC 15927) was obtained from American Type Culture Collection. Freeze-dried cells were rehydrated and cultured in Tryptic Soy Broth (TSB) and on Tryptic Soy Agar (TSA) and incubated at 37℃ for 16 h. A single colony from the agar plate was then inoculated into TSB and grown overnight. After 16 h of incubation, bacterial cells were harvested by centrifugation at 4,500 rpm for 10 min using Beckman L8-70M centrifuge and washed three times with phosphate-buffered saline (PBS, pH 7.2). The final bacterial suspension was stored at 4℃ prior to use.

### 2.3 Surface materials and characteristics

Predominant indoor materials were selected as test surfaces: flooring material (hickory wood and nylon carpet), metal (stainless steel), and glass. For the batch attachment and centrifugal detachment experiments, the surface materials were cut into 10 mm × 10 mm coupons and rinsed with deionized (DI) water and 70% ethanol to remove contaminants. For PIV experiments, the surface materials were used two ways 1) without cutting and placed within a 300 mm × 300 mm field of view (scaled room) and 2) the materials were cut into 25.4 mm × 25.4 mm coupons (pipe-flow condition). Three surface characteristics were evaluated: surface roughness, contact angle which evaluated hydrophobicity and/or hydrophilicity, and porosity.

#### 2.3.1 Surface roughness

Surface roughness was calculated in two-dimensional (2D) form. Surface micrographs of each material were obtained using a scanning electron microscope (SEM), TESCAN Vega-3 (TESCAN, CZ). The micrographs were acquired at magnifications ranging from 94 ×  to 451 ×  depending on the materials. Surface roughness parameter was expressed as the root mean square roughness (RMS), *R_q_* (*μ*m), which represents the roughness parameter in statistical method and was calculated based on Equation 1 (29), where *n* is the number of intersections of the profile and *y_i_* (μm) is the height between peak point and mean line at *i*^th^ peak.

$$R_{q}=\sqrt{\frac{1}{n}\overset{n}{\underset{i=1}{\sum }}\;y_{i}^{2}}$$ (1)

#### 2.3.2 Contact angle

Surface contact angle was measured using a VCA Optima system (AST Products, Inc., USA) to characterize the surface hydrophobicity and hydrophilicity. The instrument captures the profile of a deionized (DI) water droplet immediately after it contacts the surface material and determines the contact angle from the droplet shape. Five replicate measurements were performed for data reliability. Surfaces with contact angles below 90° were classified as hydrophilic, whereas those with contact angle above 90° were classified as hydrophobic (30).

#### 2.3.3 Porosity

Porosity was evaluated using mercury intrusion porosimetry (MIP) with a POREMASTER system (Quantachrome Instruments Corporate, USA). Mercury was forced into the surface pores under controlled pressure, and the intruded volume was recorded. Low-pressure (LP) and high-pressure (HP) were used to distinguish large and small pores, respectively. Larger pores fill at lower pressure whereas progressively higher pressures access smaller pores. For each measurement, the surface material was placed into the chamber, which was then filled with mercury, and then the pressure gradually increased to intrude mercury into the pores structure.

### 2.4 Bacterial attachment test and centrifugal detachment test

**Figure 1:**
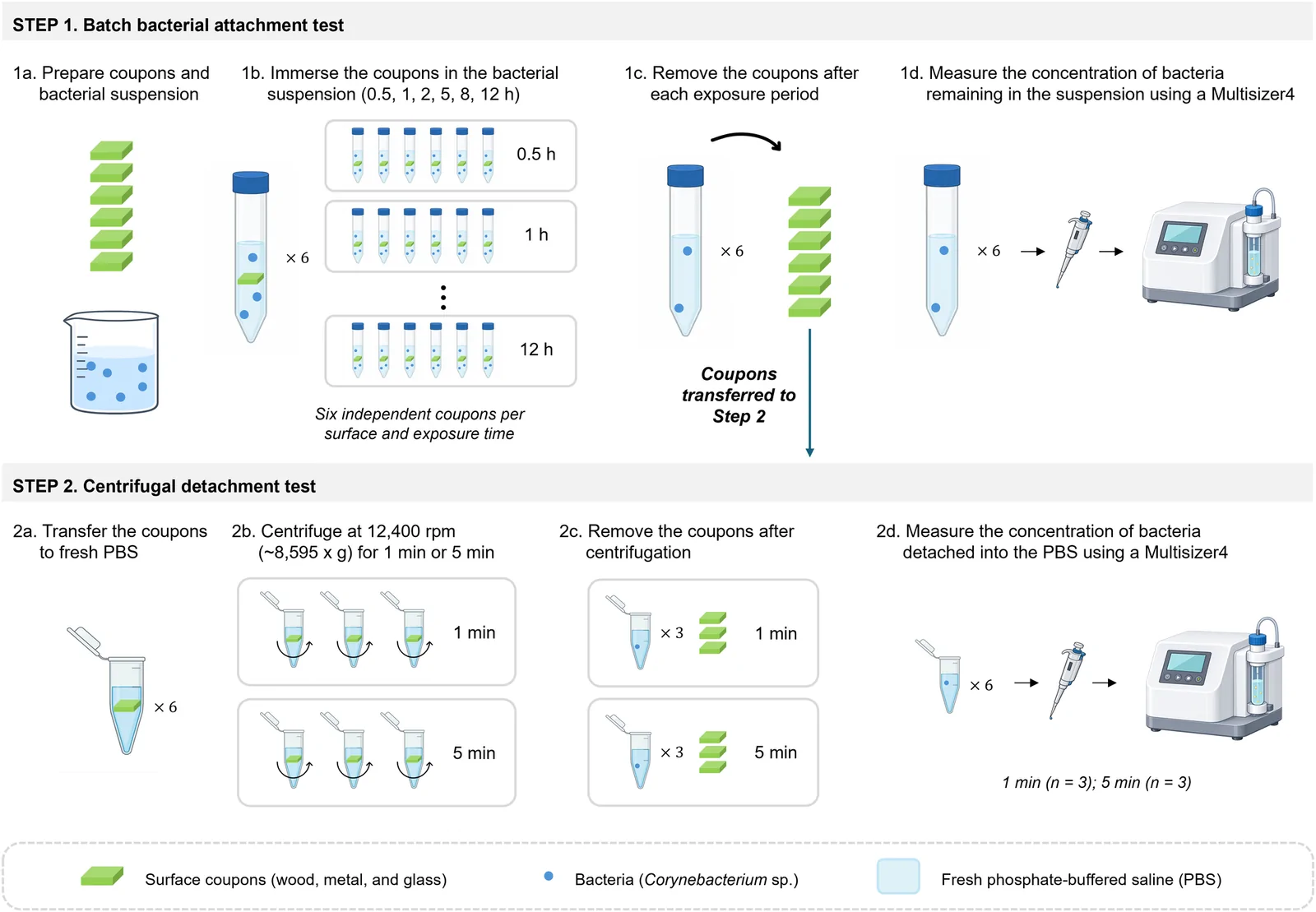
Experimental workflow for the batch attachment test (step 1) and centrifugal detachment test (step 2). For each surface and exposure period, six independent coupons were immersed in a bacterial suspension. The exposure periods were 0.5, 1, 2, 5, 8, and 12 h. After exposure, the coupons were removed and the remining suspension was analyzed using a Multisizer4. The coupons were then individually transferred to fresh phosphate-buffered saline and divided into two groups for centrifugation at 12,400 rpm (∼8,595 × g) for either 1 min (*n* = 3) or 5 min (*n* = 3). After centrifugation, the coupons were removed and the PBS containing detached bacteria was analyzed using a Multisizer4.

Experimental procedures are illustrated Figure 1. For the attachment test (Step 1), surface material coupons were immersed in bacterial suspension for 0.5, 1, 2, 5, 8, and 12 h. The bacterial suspension was prepared from the same stock and equally distributed into individual test tubes. At each exposure time, six independent coupons per surface were prepared. The number of attached bacteria was calculated by subtracting the number of bacteria remaining in suspension from the initial number of bacteria in the suspension as shown in panels 1a–1d of Figure 1. Suspended bacterial concentration was quantified using a Multisizer4 (Beckman Coulter, Inc., USA). A PBS blank was measured as a background control and subtracted from each sample count. While the approach is indirect, to obtain reliable numbers six independent replicates of the attachment test was conducted to reduce the associated uncertainty of the approach.

Bacterial detachment was subsequently assessed using a centrifugal method (Step 2), performed in three independent replicates per centrifugation duration. After the attachment test, all coupons were transferred to fresh PBS and centrifuged for either 1 min or 5 min at a fixed speed of 12,400 rpm, corresponding to a relative centrifugal force (RCF) of approximately 8,595 × g, with a rotor radius of 0.05 m, to compare bacterial detachment under different conditions. During centrifugation, adhered bacteria experience a centrifugal force directed away from the axis of rotation. Detachment occurred when this force exceeded the adhesion force between the bacteria and the surface (31). After centrifugation, the surface coupon was immediately removed from the suspension, and the suspension was mildly shaken by hand prior to sampling to resuspend any bacteria settled to the tube bottom. It is possible that the mild hand shaking may result in some bacteria remaining deposited which could contribute to a slight overestimate of the attached fraction after centrifugation. The number of detached bacteria was quantified using the Multisizer4. Only wood, metal, and glass were selected for this experiment because carpet fibers were released into the bacterial suspension during submersion, which interfered with accurate bacterial particle quantification.

### 2.5 PIV experiment

**Figure 2:**
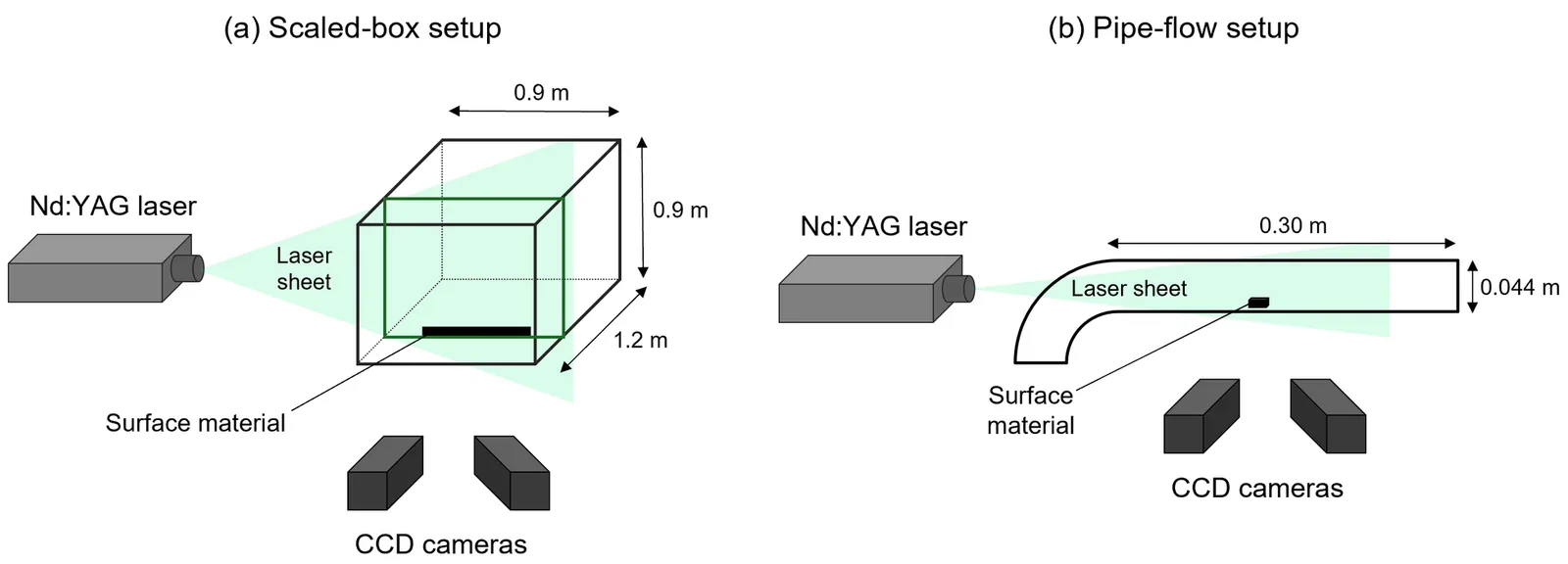
Schematic of the particle image velocimetry (PIV) experimental setups: **(a)** scaled-room system and **(b)** pipe-flow system. A Nd:YAG laser generates a planar laser sheet to illuminate particles within the measurement region. In the scaled-room system, surface materials are placed on the bottom surface of a chamber. In the pipe-flow system, surface coupons are positioned at the bottom of a pipe. Two CCD cameras are positions to capture particle movement within the field of view.

PIV experiments were conducted to visualize the bacterial particle trajectories near the surface under air-flow conditions. This provides additional insight into the pattern of bacterial particle transport over real-world surfaces in built environments. A schematic of the PIV experimental setups is illustrated in Figure 2.

Two PIV configurations were conducted to simulate indoor conditions: a scaled ventilated system to simulate an indoor space and a pipe-flow system to simulate the pipe component of an HVAC system. The scaled system measured 1.2 m in length × 0.9 m in width × 0.9 m in height. The surface materials were placed within a 0.3 m × 0.3 m field of view. In the pipe-flow experiment, a pipe with a diameter of 0.044 m was used, and flat surface coupons were placed at the center of the pipe bottom, aligned with the inner pipe wall. The gap between the flat coupon and the curved pipe wall was located outside the flow domain, so no part of the coupon protruded into the airflow. The flow was fully developed under laminar conditions. Because of the limited size of the available surface materials, only carpet, wood, and glass were tested in the scaled-room experiment, whereas carpet, wood, glass, and metal were tested in the pipe-flow experiment. Carpet as a real-surface condition for pipe-flow system simulation was used to allow comparison between the two simulation setups.

The scaled system with PIV setup was used to characterize near-surface velocity fields over larger surface areas, whereas the pipe-flow setup was used to investigate local velocity gradients very close to the surface with higher spatial resolution under a well-defined flow condition. The pipe-flow experiment simulated higher-velocity airflow conditions such as HVAC systems and investigated how direct external forces affect both bacterial particle transport to the surface and detachment under aerodynamic stress.

In each configuration, the flow field was first characterized using oil droplets generated by an oil droplet generator (Model 9307, TSI, USA) as seeding particles to establish the background velocity field. Subsequently, *Corynebacterium* sp. was aerosolized using a Collison nebulizer (CH Technologies, USA) with an aerodynamic size range of approximately 1–3 μm in diameter. These bacterial particles were tracked to obtain the near-surface velocity fields. Particle images were captured using two CCD cameras (Imager Pro X, LaVision, Germany) synchronized with a Nd:YAG laser that illuminated the measurement plane. To ensure reproducibility, each PIV measurement was repeated three times, and the reported velocity fields represent the average of these independent measurements.

### 2.6 1-D convection-diffusion model

A one-dimensional convection-diffusion model was developed to investigate the changes in bacterial concentration as a function of wall-normal distance (32). The governing equation in non-dimensional form is given below:

$$\frac{\partial N^{*}}{\partial t^{*}}+u^{*}\frac{\partial N^{*}}{\partial y^{*}}=\frac{1}{Pe}\frac{\partial^{2}N^{*}}{\partial y^{*2}}$$ (2)

where N*=N/N_0_ is the dimensionless bacterial concentration, y*=y/H is the dimensionless wall-normal distance, and t*=t/t_end_ defined with respect to an arbitrary end time *t_end_*. Here, *H* is the boundary thickness for the scaled system or pipe-flow configuration. The Péclet number is defined as Pe = *u*_y_*H/D*, where *u*_y_ is the measured near-wall velocity component and *D* is the Stokes-Einstein diffusivity of a bacterial particle. A Neumann boundary condition was applied at the wall and far field, enforcing zero normal concentration gradient (∂N*/∂y* = 0) (33). Transport was computed only in the wall-normal velocity (*u*_y_) to quantify how convection and diffusion redistribute bacterial particles away from or toward the wall over time. ‘+ *u*_y_’ represents upward flow that tends to favor resuspension, whereas ‘- *u*_y_’ represents downward flow that favors deposition toward the surface. The transport model is driven solely by the PIV-measured near-wall velocity field to provide a complementary, flow-based description of near-surface bacterial transport that parallels the attachment and detachment behavior characterized in the liquid-phase experiments.

### 2.7 Statistical analysis

All statistical analysis was performed in R 4.5.1. Because the attachment and detachment fractions were non-normally distributed, non-parametric methods (the Kruskal–Wallis test and the Scheirer-Ray-Hare test) were used throughout. The effects of surface type and attachment/detachment time on the fraction of attachment and detachment were first assessed using the one-way Kruskal–Wallis rank-sum test. To evaluate the combined and interactive effects of two factors, the non-parametric two-way Scheirer-Ray-Hare test was applied. For attachment, the model included surface and contact time as factors. For detachment, the models included surface × contact time and surface × centrifugal detachment time. A significance threshold of *α* = 0.05 was applied to all tests.

## 3 Results

### 3.1 Surface properties

Surface roughness, porosity, and contact angle were measured to characterize the surface topography, pore structure, and wettability of the indoor surface materials. Table 1 summarizes the measured surface roughness, porosity, and contact angle values. Surface roughness was quantified as the root mean square roughness (*R_q_*) obtained based on the SEM profiles. Carpet exhibited the highest roughness (256.96 μm), indicating a highly textured surface compared with the other materials. Among the non-fibrous materials, wood (hickory) showed the highest roughness (46.36 μm), while metal (stainless steel) and glass exhibited relatively smooth surfaces (12.04 μm and 6.15 μm, respectively). Porosity was measured using MIP to investigate the contribution of both large and small pores. Wood had a porosity of 10.25%. Carpet showed the highest porosity (22.86%), whereas the total porosities of metal and glass were below 1% indicating that it behaved as a non-porous substrate. Contact angle was measured to assess the surface wettability based on the 90° criterion. Wood and glass were classified as hydrophilic, whereas metal was the most hydrophobic surface. Glass had the lowest contact angle (22.75°), hence, the most hydrophilic surface. Wood (63.02°) showed intermediate wettability among the tested materials. The contact angle of carpet could not be measured because its rough, fibrous surface topography prevented clear determination of droplet shape.

**Table 1:** Surface roughness (R_q_), porosity (low, high, total), and water contact angle of the tested indoor surface materials.

Surface materials	Roughness, R_q_ (μm)	Porosity (%)			Contact angle (°)
		Low	High	Total	
Wood (Hickory)	46.36	10.25	ND	10.25	63.02 ± 9.66
Metal (Stainless-steel)	12.04			<1^a^	103.05 ± 14.05
Glass	6.15			<1^a^	22.75 ± 3.55
Carpet	256.96	15.23	7.63	22.86	N.D.^b^

a Literature value, MIP measurement not applicable for non-porous materials (50).

b N.D., no data.

### 3.2 Bacterial attachment and detachment

#### 3.2.1 Attachment experiment

*Corynebacterium* sp. attachment on indoor surface materials was monitored over a 12-hour period. Adhesion was evaluated at 0.5, 1, 2, 5, 8, and 12 h, and detachment was assessed after centrifugal treatment for 1 min and 5 min at each attachment interval.

**Figure 3:**
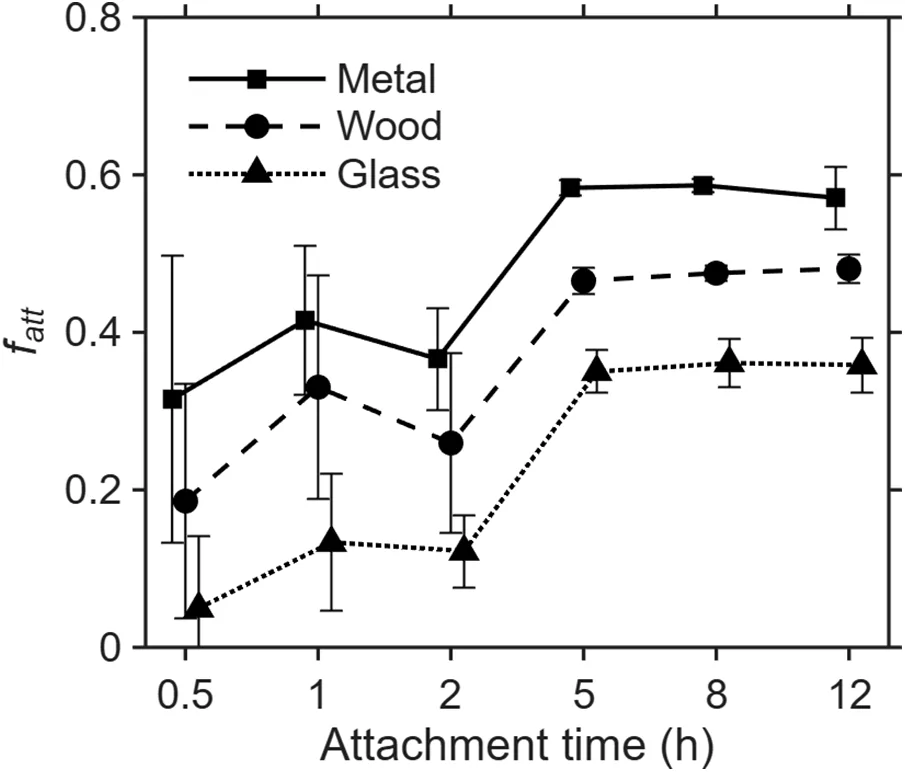
Attachment fraction (*f_att_*) of particles on metal, wood, and glass surfaces as a function of attachment time. Error bars represent ± SD (*n* = 6).

Figure 3 shows the attachment fraction (*f*_att_ = *N*_attached_/*N*_initial_) as a function of attachment time for three surface materials. The attachment fraction represents the ratio of attached bacteria to the initial number of suspended bacteria. All surfaces showed increasing attachment fractions with longer exposure times, indicating that prolonged contact provided more opportunity for bacteria-surface bindings. Among the tested materials, metal showed the highest attachment fraction (∼0.59), followed by wood (∼0.48) and glass (∼0.36). The Kruskal–Wallis test showed that the type of surface was significant for both bacterial attachment (*p* < 0.001) and detachment (*p* = 0.0117), while attachment time was significant only for bacterial attachment (*p* < 0.001). The Scheirer-Ray-Hare test indicated that the surface × attachment time interactions were not significant for either fraction. All *p*-values were summarized in Table 2.

**Table 2:** Summary of statistical analysis for bacterial attachment (*f_att_)* and detachment *(f_det_)* fractions. Values are *p*-values from the one-way Kruskal–Wallis test (top) and Scheirer-Ray-Hare test (bottom).

Kruskal–Wallis test					
Factor	Surface	Attachment time	Detachment time		
Attachment, *f_att_*	9.64 × 10^−6^	1.15 × 10^−5^	–		
Detachment, *f_det_*	0.0117	0.4750	0.8293		

Scheirer-Ray-Hare test					
Factor	Surface	Attachment time	Detachment time	Surface × Attachment time	Surface × Detachment time
Attachment, *f_att_*	0.0000	0.0000	–	0.9999	–
Detachment, *f_det_*	0.0116	0.4723	0.7908	0.7065	0.0093

#### 3.2.2 Centrifugal detachment experiment

**Figure 4:**
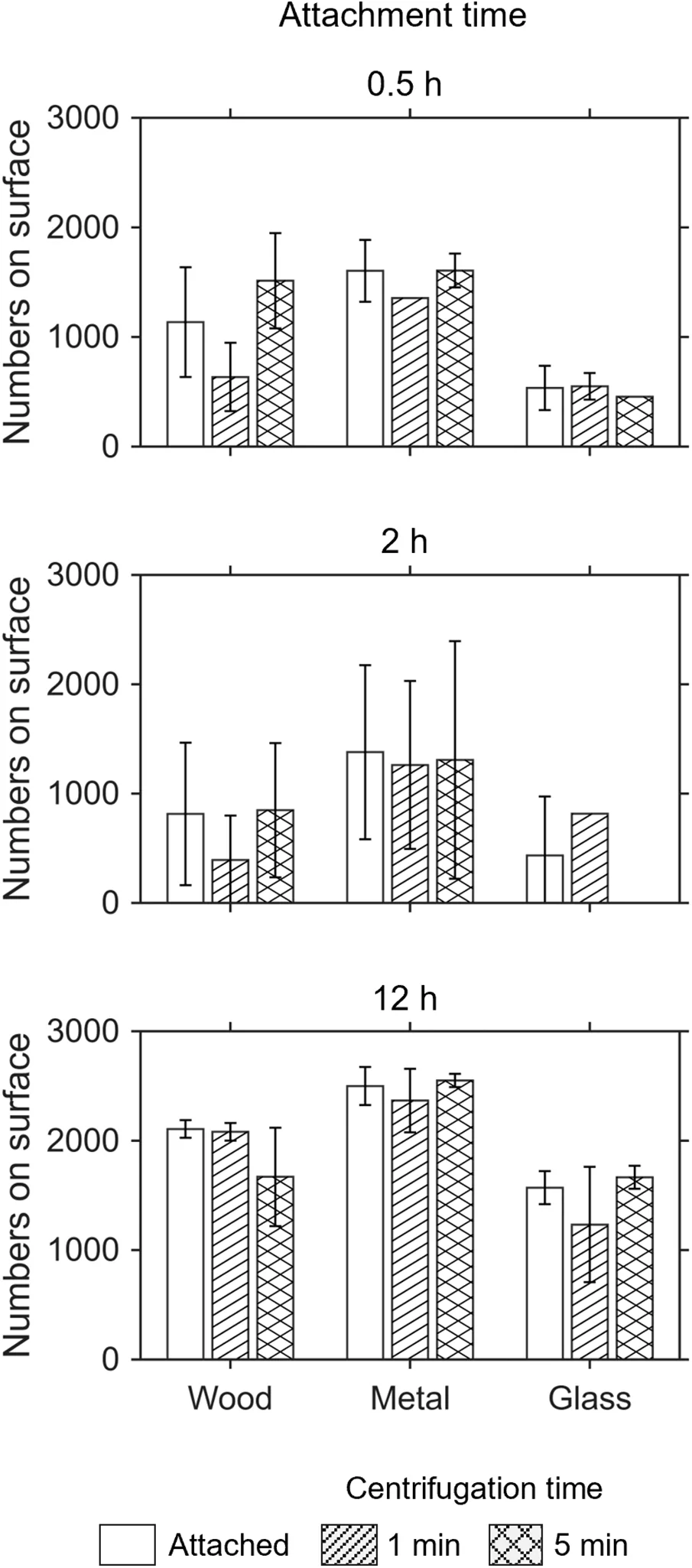
The number of bacterial particles on wood, metal, and glass at representative attachment times. Solid bars indicate the number of attached bacteria; hatched bars indicate particles remaining after 1 min or 5 min of centrifugal detachment. Error bars represent ± SD (*n* = 6 for attachment, *n* = 3 for detachment).

The numbers of bacteria on wood, metal, and glass surfaces at representative attachment times and after each detachment treatment are shown in Figure 4. At the early stage of attachment (0.5 h), bacteria adhered predominantly through reversible interactions. The large differences between the attached and post-detachment bars indicate that bacteria were easily removed after both one minute and five minutes of centrifugal detachment. Notably almost no bacteria remained on glass after five minutes of centrifugal detachment, indicating that early adhesion on glass was almost entirely reversible.

After two hours of attachment, the differences between the attached and post-detachment bars became smaller, indicating that adhesion bonds were progressively strengthened. Nevertheless, a fraction of bacteria was still removed by centrifugal detachment, as both reversible and less-reversible adhesion coexisted during this transitional phase. After 12 h of attachment, nearly similar numbers of attached bacteria before and after centrifugal detachment indicate that bacterial adhesion gradually shifted toward a less reversible state on all three surfaces as exposure time increased.

Therefore, at 0.5 and 2 h, the differences in the number of bacteria remaining on the surface after centrifugation indicate that centrifugal detachment had effect during early attachment stages. At 12 h, however, the numbers of bacteria remaining on the surface after both 1 min and 5 min centrifugation were similar to the initially attached counts, showing that adhesion had become less reversible with prolonged contact. Glass tended to show slightly greater detachment compared to wood and metal, likely reflecting weaker adhesion on its smooth, low-roughness surface. Differences in the total number of attached bacteria among the surface materials mostly disappeared with increasing attachment time.

These results were supported by statistical analysis (Table 2). The Kruskal–Wallis test confirmed that the surface type significantly influenced the detachment fraction (*p* = 0.0117), whereas attachment time did not have a statistically significant effect on detachment (*p* = 0.475). Detachment tended to decrease with prolonged contact time, however this trend did not reach statistical significance, likely attributable to the high variability and the high proportion of near-zero values in the strongly attached samples (34). The Scheirer-Ray-Hare test showed a significant surface × centrifugal detachment time interaction (*p* = 0.0093), indicating that the effect of detachment duration (1 vs. 5 min) differed among the surfaces, consistent with the surface-dependent differences described in Figure 4.

### 3.3 PIV measurements

**Figure 5:**
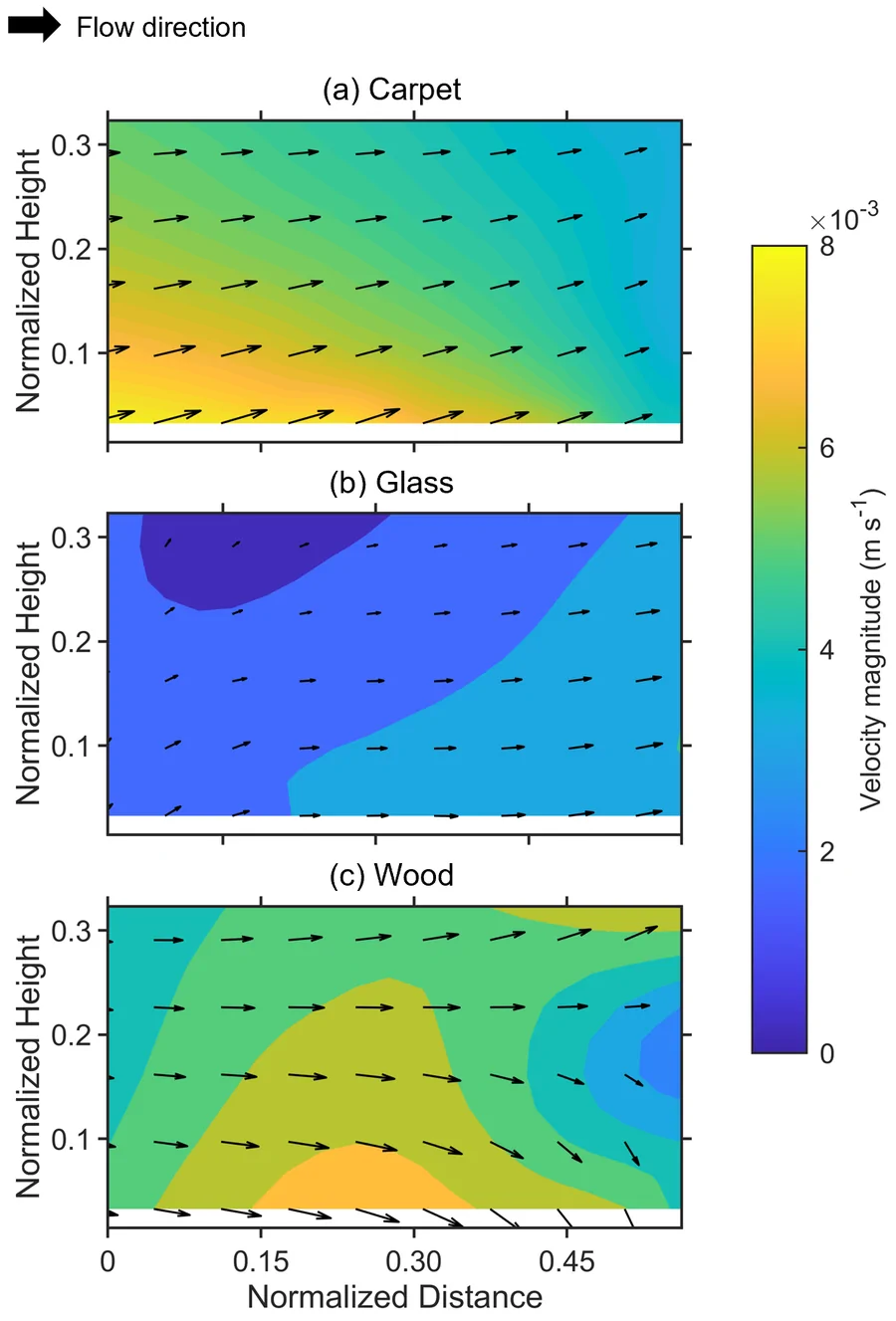
Velocity magnitude fields measured by PIV in a scaled-room setup for three surface materials: **(a)** carpet, **(b)** glass, and **(c)** wood. Black arrows indicate local flow direction. Axes are normalized by the test section height.

Scaled-room PIV measurements were conducted to characterize the near-surface velocity fields over carpet, glass, and wood, and the results are shown in Figure 5. Metal was not included in the scaled-room PIV experiment due to limited availability of the same metal type required for consistent comparison. The distance was normalized by the test-section height. Distinct surface-dependent flow patterns were observed. Near the carpet surface (Figure 5a), the velocity vectors were relatively parallel to the surface with a slight upward inclination toward the right, with higher velocity magnitudes observed in the lower region close to the surface, decreasing gradually with increasing downstream distance. Near the glass surface (Figure 5b), the flow direction was largely parallel to the surface, and the gradual transition from near-zero velocity to higher values toward the right indicates that the flow developed slowly over the glass surface consistent with its smooth and low-roughness character. Despite having lower mean roughness than carpet, the wood surface (Figure 5c) showed the most complex flow pattern with higher velocity in the central region and relatively lower velocities on both sides. In the central region, the flow was directed rightward and approximately parallel to the surface, whereas in the right region, the velocity vectors were inclined toward the surface. This spatial variation indicates a more heterogeneous near-wall flow structure over wood compared to the more uniform flow over carpet.

**Figure 6:**
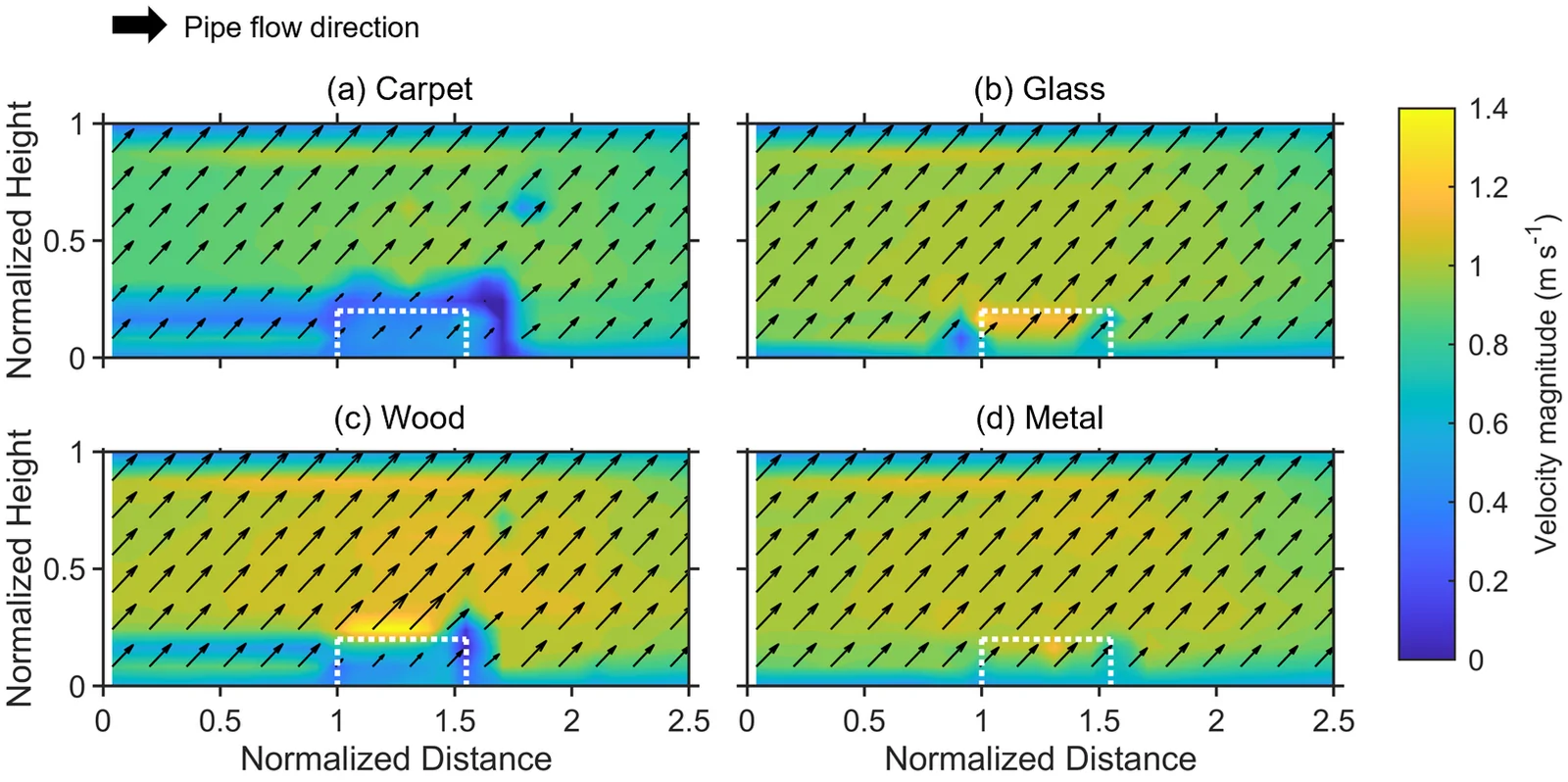
Velocity magnitude fields measured by PIV in the pipe-cross section for four surface materials: **(a)** carpet, **(b)** glass, **(c)** wood and **(d)** metal. Black arrows indicate the flow direction and relative magnitude. White dash lines indicate the edges of the surface coupons. Axes are normalized by the pipe diameter. Flow direction is indicated by the arrow at the top.

To gain more insight into the micro-environments at the surface, PIV experiments were conducted in the pipe-flow condition, as shown in Figure 6, where the white dashed boxes indicate the surface material locations. In the pipe-flow experiment, the overall flow direction was downstream towards the right. However, distinct differences were observed in the near-surface region of the coupons highlighted by the dashed boxes. Over the carpet surface, a lower-velocity region was observed near the surface, while glass and wood showed higher near-wall velocities. For glass, the lower velocity region was more confined, and a localized high velocity was observed immediately above the surface sample. Wood exhibited the higher velocity near the surface and a broader low-velocity region near the pipe bottom, indicating greater spatial heterogeneity in the near-wall flow structure. Metal exhibited the most uniform near-wall flow field among the tested materials, with minimal disturbance around the metal surface. These results demonstrate that each surface material generated a distinct near-surface environment, which may influence bacterial particle transport, deposition, and detachment under airflow conditions.

**Figure 7:**
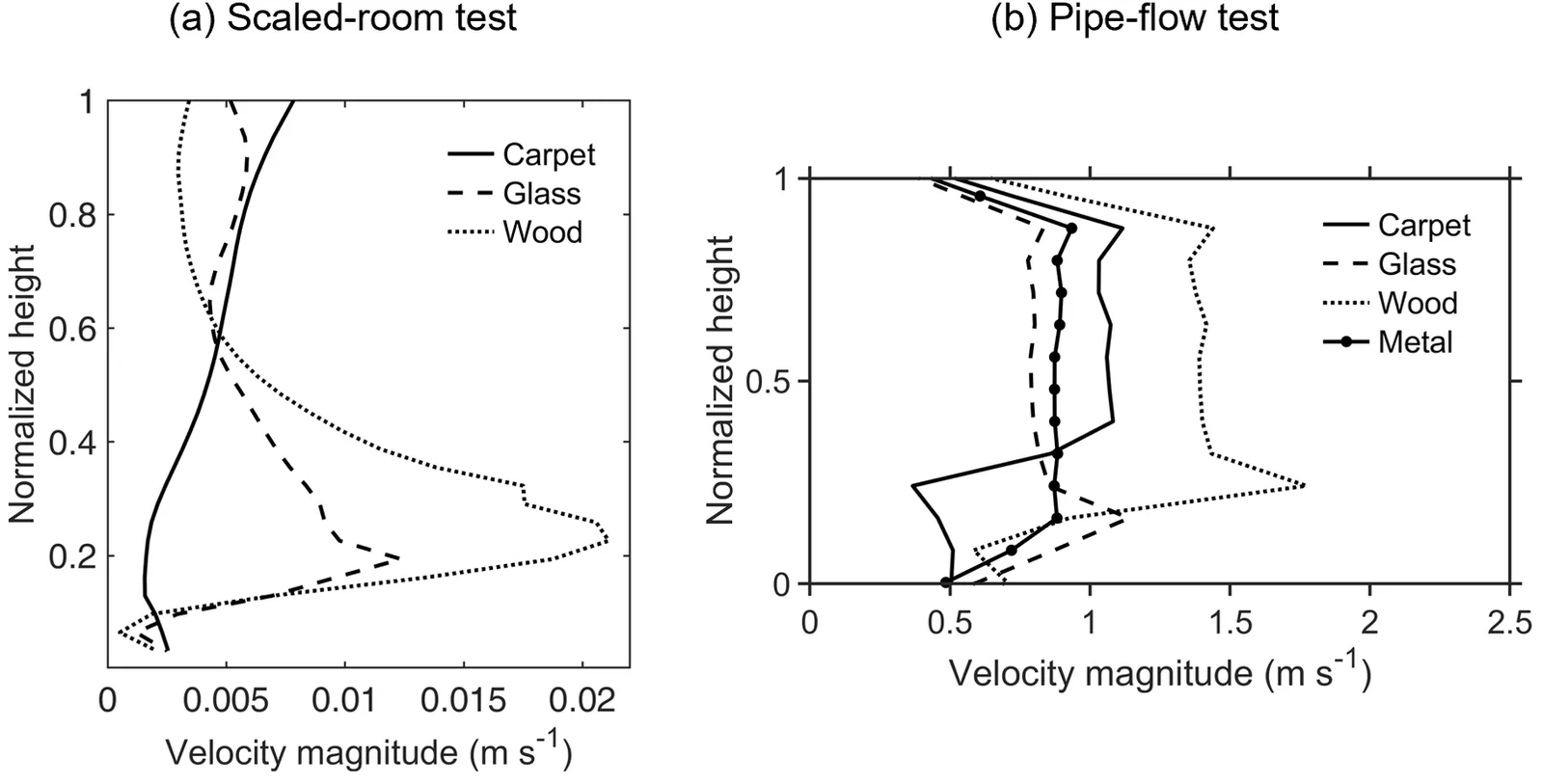
Vertical profiles of velocity magnitude for different surface materials under **(a)** scaled-room test at *x*/*H* = 0.3 and **(b)** pipe-flow test at *x*/*D* = 1.25. Velocity magnitude is plotted as a function of normalized height (*y*/*H* or *y*/*D*). Different line styles represent surface materials (carpet, glass, wood and metal).

The comparison between the scaled-room PIV experiment (Figure 5) and the pipe-flow PIV experiment (Figure 6), together with their corresponding velocity profiles (Figure 7) provides complementary aspects of surface-dependent conditions relevant to bacterial particle attachment and detachment in indoor air environments. The scaled-room experiment represents conditions similar to gentle air circulation in indoor spaces, where surface materials create distinct near-surface velocity gradients that influence the initial approach and deposition of airborne bacterial particles. The velocity magnitude profiles at a normalized location of *x*/*H* = 0.3 (Figure 7a) show that wood and glass exhibit relatively higher velocities near the surface with steeper gradients, whereas carpet displays a more gradual increase in velocity with height. The velocity profiles under pipe-flow conditions (Figure 7b) indicate that wood maintains higher near-surface velocities, while glass and metal show more uniform profiles with minimal disturbance, indicating reduced flow resistance near the surface.

The quantitative near-surface fluid characteristics from both PIV experiments are summarized in Table 3. The near-surface Reynolds numbers ranged from approximately 146–4,023 across the two configurations. In the scaled-room experiment, the flow remained laminar (Re < 600), whereas in the pipe-flow experiment, Re (940–4,023) corresponded to the transitional regime, where typical HVAC flows generate near-surface shear that drives particle transport. The mean wall shear stress on glass was almost identical between the two PIV experiments despite the large difference in near-surface velocities (0.004 vs. 1.034 m/s). Carpet consistently exhibited the highest shear stress across both experiments unlike glass and wood surfaces. This reflects the flow-regime-dependent behavior of surface materials under different flow conditions.

**Table 3:** Near-surface fluid characteristics for different surface materials in scaled-room and pipe-flow PIV experiments.

Type	Fluid characteristics	Carpet	Glass	Wood	Metal
Scaled-room^a^	Near-surface velocity, u_s_ (m s^−1^)	0.007	0.004	0.006	N.D.^c^
	Reynolds number near surface, Re	133.06	73.04	116.46	N.D.
	Mean wall shear stress, $\left|{\bar{\tau }}_{w}\right|$ (Pa)	7.47 × 10^−7^	4.28 × 10^−7^	2.25 × 10^−7^	N.D.
	Peclet number, Pe (*H*^d^ = 0.0045 m)	6.39 × 10^5^	3.51 × 10^5^	5.60 × 10^5^	N.D.
Pipe-flow^b^	Near-surface velocity, u_s_ (m s^−1^)	0.325	1.034	1.390	1.036
	Reynolds number near surface	939.65	2993.44	4022.84	2998.56
	Mean wall shear stress, $\left|{\bar{\tau }}_{w}\right|$ (Pa)	4.90 × 10^−6^	5.11 × 10^−7^	2.28 × 10^−6^	7.35 × 10^−7^
	Peclet number, Pe (*H*^d^ = 0.0017 m)	1.16 × 10^7^	3.70 × 10^7^	4.98 × 10^7^	3.71 × 10^7^

Mean wall shear stress was estimated from near-wall velocity gradients using the slope method (${\bar{\tau }}_{w}$=*μ*∂*u*/∂*y*).

a Measurement region: normalized distance 0.04–0.14.

b Measurement region: normalized distance 1.15–1.55.

c N.D. No Data.

d *H* indicates the boundary thickness.

### 3.4 1-D convection-diffusion modeling

**Figure 8:**
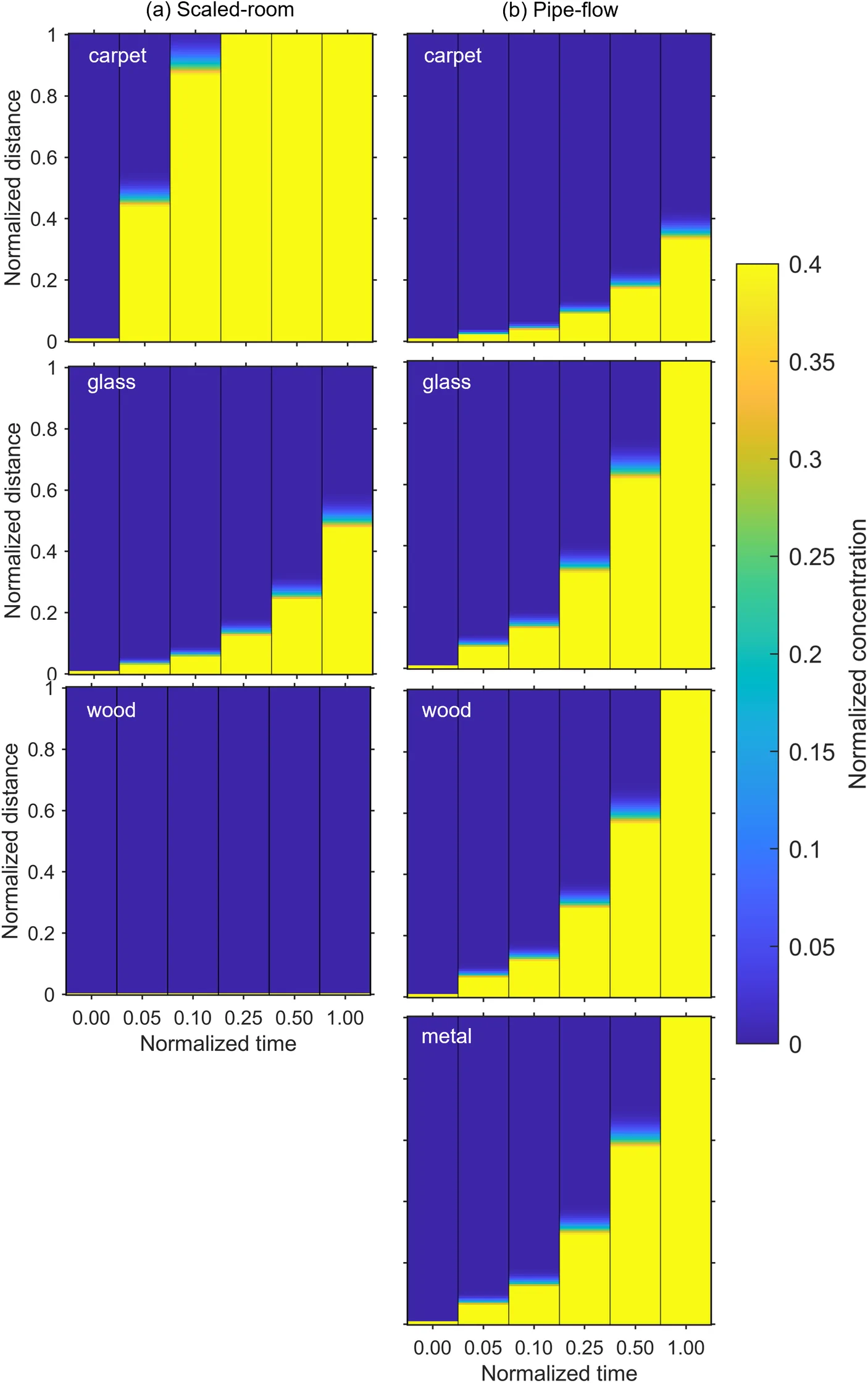
Spatiotemporal evolution of normalized bacterial concentration profiles predicted by 1-D convection-diffusion model for **(a)** scaled-room and **(b)** pipe-flow scenarios over indoor surface materials (carpet, glass, and wood for scaled-room; carpet, glass, wood and metal for pipe-flow), respectively. The *x*-axis represents normalized time (t*=*t*/*t_end_*) and the *y*-axis indicates normalized distance from wall (y*=*y*/*H*), where *H* is the characteristic domain height and y* = 0 corresponds to the wall (surface) while y* = 1 corresponds to the far field. The normalized concentration (N*=*N*/*N_0_*) is defined relative to the initial concentration *N*_0_ and ranges from 0 (y* = 1, far field) to 1 (y* = 0, wall boundary) at *t* = 0. Zero gradient Neumann boundary conditions were imposed at both the wall and the far field. Wall-normal velocity components (*u_y_*) obtained from PIV measurement were used as model inputs for each surface.

The one-dimensional convection-diffusion model described by Equation 2 was used to predict the spatiotemporal evolution of normalized bacterial concentration profiles on surface materials. Figure 8 shows the dimensionless concentration fields as a function of normalized time and normalized distance from the wall. Zero-gradient Neumann boundary conditions were imposed at both the wall and the far field, so that no diffusive flux occurs at either boundary.

In the scaled-room (Figure 8a), carpet showed rapid upward transport from the wall, whereas glass showed comparatively slower upward spreading. Wood showed no upward transport because the PIV-measured wall-normal velocity was negative (*u*_y_ < 0), directing bacterial particles toward the wall and showing a deposition-favored pattern. In contrast, under pipe-flow conditions (Figure 8b), all surfaces showed progressive upward transport, but carpet showed slower concentration transport than other surfaces due to its lower near-wall velocity. Glass, wood, and metal showed similar upward transport rates across all time steps. Since the Péclet numbers (Pe) were on the order of 10^5^–10^7^ for both scaled-room and pipe-flow conditions, convective transport strongly dominates over diffusion at the near-surface flow scale. For determining Pe, the boundary layer thickness (*H*) was taken as the characteristic length scales for scaled-system PIV and pipe-flow PIV (0.0045 m and 0.0017 m, respectively), whereas the Reynolds numbers were based on the system geometric length scales (0.3 m and 0.044 m, respectively). Accordingly, the concentration profiles in Figure 8 are primarily generated due to the direction and magnitude of the wall-normal velocity (*u*_y_).

## 4 Discussion

The present study investigated the temporal microbial attachment and detachment behaviors on representative indoor surfaces and examined how these processes are influenced by near-surface airflow conditions. The findings demonstrated that early-stage bacterial attachment was strongly affected by surface properties, whereas prolonged exposure promoted stronger and a gradual shift toward a less reversible state, regardless of surface type under tested condition. Near-surface boundary conditions further influenced bacterial particle transport and detachment behavior, although their effects varied depending on the flow configuration and airflow intensity.

### 4.1 Attachment and detachment behaviors

The present study showed that microbial attachment increased with exposure time, whereas no statistically significant effect of centrifugation duration on bacterial detachment was detected under the tested conditions. These findings suggest that longer attachment periods may have promoted progressive stabilization of bacteria-surface interactions, and gradual shift toward a less reversible state. However, because attachment and detachment were estimated indirectly from suspension measurements and only three coupons were tested for each centrifugation duration, the present results cannot be considered conclusive evidence of irreversible adhesion. In addition, 12 h was the longest exposure time evaluated, and adhesion behavior beyond this period remains unknown. A slight decrease in *f_att_* observed at 2 h is attributed to the dominance of spontaneous detachment over new attachment during an unstable reversible adhesion phase, in which loosely adhered bacteria can return to the bulk suspension without external force. This time-dependent transition from reversible to progressively stable (irreversible) adhesion has been widely reported in previous studies (35–37). Single-cell tracking study has confirmed that the transition from an initial reversible phase to a stronger, irreversible adhesion state can occur within seconds to minutes once surface contact occurs (37). Following initial surface contact, adhesion bonds progressively strengthen, and substantially greater force is required for bacterial removal than for preventing initial attachment (19). In our experiments, *Corynebacterium* sp., a non-motile, non-flagellated organism that produces fimbriae, showed increasing attachment fraction with exposure time across all tested surfaces. This trend is compatible with prior work indicating that initial adhesion is mediated by weak, fimbriae-facilitated surface contact (38), followed by adhesion bond strengthening through cell wall deformation and extracellular matrix production for biofilm development (32). These findings suggest that surface properties and bacterial characteristics are most important during the initial attachment stage, whereas prolonged contact promotes stronger adhesion and potential physical retention that reduce bacterial detachment.

Surface wettability in our study also influenced *Corynebacterium* sp. attachment and detachment behavior. We observed the highest attachment fraction on metal likely due to its greater hydrophobicity and largest contact angle (103.05°), consistent with previous report that moderately hydrophobic surfaces with contact angle near 90° favor bacterial adhesion (18). However, bacterial attachment was likely governed by a combination of hydrophobicity, low porosity, and surface chemistry as shown by other studies (23).

The attachment and detachment behaviors can also be interpreted using extended Derjaguin-Landau-Verwey-Overbeek (XDLVO) theory. Increased surface hydrophobicity can promote attractive acid–base interactions and enhance bacterial attachment (39, 40), which may explain the greater attachment observed on metal surfaces in our experiments. In contrast, the strong hydrophilicity of glass likely increase repulsive interactions and reduce bacteria–surface contact probability, consistent with the lower bacterial adhesion observed on glass (41). However, the XDLVO framework should be applied cautiously because the XDLVO theory assumes ideal, homogeneous surfaces, whereas the tested indoor surfaces were heterogeneous in physicochemical composition (40).

### 4.2 Near-surface airflow and material-dependent mechanics

The PIV results demonstrated that surface materials strongly influence near-surface flow structures, which affect airborne particle transport, deposition, and potential resuspension. Carpet likely created two distinct near-surface regions or micro-environments: an outer-fiber region that interacted directly with airflow and an inter-fiber region where bacterial particles may experience greater retention. Higher velocities above the carpet surface suggest that fibrous roughness enhanced near-surface mixing compared with smoother materials (42, 43). In contrast, very low velocities close to the fiber surface indicate that the region is favorable for the retention of bacterial particles.

Glass surface exhibited the lowest flow disturbance and more uniform near-wall velocity development because of its smooth and low-roughness character (44). Although wood had lower mean roughness than carpet, it showed the most spatially heterogeneous flow pattern, indicating that its irregular topography and pore structure generated localized flow deviations or recirculation zones near the surface (45).

In the pipe-flow model, the near-surface Reynolds numbers reflect the near surface shear that drives particle transport by HVAC-like flows, whereas the scaled-room remained in the laminar regime. Therefore, in scaled-room, the observed material-dependent velocity differences are more likely associated with roughness-induced modifications of local velocity gradients and near-wall recirculation rather than turbulence generation (44, 46).

### 4.3 Peclet number analysis and 1-D convection-diffusion modeling

The Péclet numbers (Pe) provide a physical basis for relating the liquid-phase centrifugal detachment experiments to air-phase particle transport under indoor airflow conditions. Both the PIV and centrifugal detachment experiments exhibited advection-dominated transport regimes (Pe ≫ 1) (47). Both PIV experiments showed substantially higher Pe values (up to ∼4.98 × 10^7^) demonstrating a strongly advection-dominated regime in which near-surface shear stress governs particle transport and diffusive processes are negligible. These large Pe values are consistent with the elevated near-surface Re reported in Table 3. For aerosolized bacteria, even modest airflows generate large Pe values due to their very low Brownian diffusivity (48), so advection-dominated transport is expected in indoor surface environments.

The one-dimensional convection–diffusion model further demonstrated how near-wall flow structures create either resuspension-favored or deposition-favored conditions. Positive wall-normal velocity components (*u_y_* > 0) promoted upward bacterial particle transport away from the surface, whereas negative wall-normal velocity (*u_y_* < 0) over wood under scaled-room conditions directed bacterial particles toward the wall and favored deposition. Under pipe-flow conditions, carpet showed slower upward transport than the other surfaces because its relatively low near-wall velocity and fibrous structure likely limited airflow penetration into the inner fiber region.

Comparison of the scaled-room and pipe-flow configurations highlights the importance of geometry-dependent airflow in controlling bacterial particle transport. Carpet retained bacterial particles within its fibrous structure, although wall-normal recirculation under scaled-room conditions enhanced upward transport from the surface. Previous studies have similarly shown that carpets can function as particle sinks while also serving as potential resuspension sources under airflow and occupant activity (49). In contrast, glass showed upward transport under both flow conditions, with faster transport occurring under higher-shear pipe-flow conditions.

### 4.4 Limitations

Overall, the liquid-phase and air-phase analyses in this study are complementary. The attachment and detachment experiments identify which surface strongly retains bacteria, while the PIV-based transport model shows how the near-surface airflow influences bacterial transport or resuspension. Several limitations of this study should be acknowledged.

First, a single bacterial species (*Corynebacterium* sp.) was used as a model organism. Because bacterial characteristics vary among the species, the attachment, detachment and transport behavior observed here may differ for other microorganisms due to differences in surface characteristics, mobility, size, or morphology.

Second, attachment and detachment were estimated indirectly from differences in bacterial concentrations measured in the suspensions rather than by direct measurements on the coupon surfaces. As a result, the calculated values may be influenced by variability in initial attachment, sample handling, and particle-count measurements. In addition, the use of only three independent coupons for each centrifugation duration may have limited ability to detect small duration-dependent differences.

Third, the detachment datasets contained some portion of zero values which reduced the statistical power of the comparisons. Although non-parametric tests (Kruskal–Wallis test and Scheirer-Ray-Hare test) were applied, some material dependent trends are discussed as observed tendencies rather than formally significant differences.

Fourth, surface porosity was characterized by MIP for the porous materials, whereas literature-based values were used for non-porous materials (glass and metal). This approach provides useful relative comparisons but does not fully capture the micro-scale topography of the tested materials that may influence bacterial behavior near the surface.

Fifth, the study was conducted under controlled laboratory conditions allowing detailed insight of controlled parameters. Field measurements were not conducted. The mechanistic findings can be informative for estimating infection or exposure risk but within the context of the study.

Finally, the one-dimensional convection-diffusion model is a representation of near-wall transport and is not a resuspension model. It does not resolve three-dimensional flow structure, particle-surface force, or turbulent fluctuation.

## 5 Conclusions

Batch attachment and centrifugal detachment tests showed that bacterial attachment increased with exposure time, while the detachment fraction was decreased. These results highlight a time-dependent transition from reversible to less reversible adhesion. Early-stage attachment was related to surface characteristics, but these material-dependent differences became diminished as exposure time increased and adhesion bonds strengthened.

The PIV measurements and convection-diffusion model predictions further demonstrated that particle resuspension is governed by the balance between near-surface forces and surface-particle adhesion. Particles deposited on exposed carpet fibers were more readily resuspended by airflow, whereas particles embedded deeper within the fibrous structure were likely more resistant to removal. In contrast, smooth surfaces generated less flow disturbance and more predictable near-wall conditions, making particle deposition and resuspension behavior easier to estimate and control. Conversely, rough and porous surfaces such as carpet and wood created heterogeneous flow environments with localized low-velocity regions that enhanced particle retention while also promoting resuspension under certain airflow conditions.

The findings suggest that near-surface airflow structure should be considered together with material properties when evaluating contamination control strategies and selecting indoor surface materials for high-contact environments such as childcare facilities. These findings have practical implications for surface material selection in high-contact indoor areas or high-activity environments. Smooth, non-porous surfaces may facilitate more effective cleaning and disinfection compared with rough, porous surfaces, thereby reducing the frequency of intensive cleaning cycles and potentially lowering infection risk.

Overall, the results indicate that near-wall airflow structure strongly influences bacterial transport, deposition, and resuspension over indoor surfaces. Rough and porous materials may generate sheltered low-velocity regions that enhance particle retention, whereas smoother surfaces generate more uniform near-wall flow conditions that may facilitate particle removal and improve predictability of deposition and resuspension behavior. For a given surface material, particle transport is strongly influenced by geometry-dependent near-wall flow structure, which can either enhance or suppress bacterial transport.

## Data Availability

The original contributions presented in the study are included in the article/Supplementary Material, further inquiries can be directed to the corresponding author.
